# Involving people with type 2 diabetes in facilitating participation in a cardiovascular screening programme

**DOI:** 10.1111/hex.13228

**Published:** 2021-03-24

**Authors:** Marie Dahl, Susanne Friis Søndergaard, Axel Diederichsen, Jens Søndergaard, Trine Thilsing, Jes S. Lindholt

**Affiliations:** ^1^ Vascular Research Unit Department of Surgery Regional Hospital Central Denmark Viborg Denmark; ^2^ Department of Clinical Medicine Aarhus University Denmark; ^3^ Centre for Research in Clinical Nursing School of Nursing Regional Hospital Central Denmark/VIA University College Viborg Denmark; ^4^ Department of Public Health, Nursing Aarhus University Aarhus Denmark; ^5^ Department of Cardiology Odense University Hospital Odense Denmark; ^6^ Research Unit for General Practice Department of Public Health University of Southern Denmark Odense Denmark; ^7^ Department of Cardiothoracic and Vascular Surgery Odense University Hospital Odense Denmark; ^8^ Elitary Research Centre of Individualized Medicine in Arterial Disease (CIMA), Odense University Hospital Odense Denmark; ^9^ Cardiovascular Centre of Excellence in Southern Denmark (CAVAC), Odense University Hospital Odense Denmark

**Keywords:** cardiovascular disease, codes of ethics, patient and public involvement, screening, type 2 diabetes

## Abstract

**Background:**

Knowledge is lacking about how to increase uptake among people with type 2 diabetes (T2D) invited to preventive initiatives like cardiovascular screening.

**Aim:**

To explore how to improve participation of people with T2D in cardiovascular screening using patient and public involvement (PPI).

**Methods:**

Patient and public involvement was included in a qualitative research design. From April to October 2019, we invited 40‐ to 60‐year‐old people with T2D (n = 17) to individual consultative meetings, using an interviewing approach. Before the interviews, participants were asked to read a proposed invitation letter to be used in a cardiovascular screening programme. Inductive content analysis was undertaken.

**Results:**

Participants considered cardiovascular screening important and beneficial from both a personal and social perspective. We found that the relational interaction between the person with T2D and the health‐care professional was key to participation and that nudging captured through the design of the screening programme and the wording of the invitation letter was requested.

**Conclusion:**

In preventive initiatives perceived as meaningful by the invitee, a focus on recruitment is crucial to facilitate participation. This study contributed with knowledge about how to promote participation by involving health‐care professionals in recruitment initiatives and through nudging. This knowledge may assist researchers, policymakers and ethicists' understanding and assessment of the ethical appropriateness and public acceptability of nudging in cardiovascular screening.

**Patient or public contribution:**

By consulting 17 people with T2D, we are now in a position to suggest how a screening initiative should be altered because tools to improve uptake have been identified.

## 1 BACKGROUND

Knowledge is lacking about how to achieve high participation rates among people with type 2 diabetes (T2D) invited to preventive initiatives like cardiovascular screening. According to the WHO, the screening threshold for participation rates should exceed 70% to ensure screening effectiveness.^1^ However, in the pilot study of the DIAbetic CArdioVAscular Screening and intervention trial (DIACAVAS) targeting people with T2D, the participation rate was only 41%. The DIACAVAS trial was designed to identify unprotected subclinical cardiovascular diseases (CVD) among 40‐ to 60‐year‐old people with T2D in Denmark in order to offer them individualized treatment.^2^ Overall, the low DIACAVAS uptake indicates that it did not meet the invitees' needs and preferences. Thus, the DIACAVAS investigators decided to strengthen recruitment by involving patients before conducting a large‐scale trial.^2^ In line with DIACAVAS, non‐participation in population‐based cardiovascular screening and health check have also been found to be associated with diabetes.^3^, ^4^

Patient and public involvement (PPI) is an acknowledged approach to improve the quality and relevance of health‐care research. It refers to ‘research being carried out “with” or “by” members of the public rather than “to,” “about” or “for” them’.^5^ PPI has been found beneficial in several ways. A systematic review and meta‐analysis by Crocker et al^6^ found that PPI interventions increased participant recruitment by 16% on average. Additionally, the likelihood of participation was three times higher when PPI interventions were conducted after having consulted people with lived experience of the condition being studied compared with people not having the targeted condition. Moreover, PPI was shown to be beneficial by improving the study design, study materials and the readability of patient information,^7^, ^8^ and by incorporating research objectives relevant to the study population.^8^, ^9^, ^10^ Therefore, the aim of this study was to explore how to improve participation in a cardiovascular screening programme among people with T2D by use of PPI.

## 2 METHODS

We employed PPI in a qualitative research design using an interviewing approach. We consulted people with T2D by conducting individual, elaborating one‐off meetings to get their views on how to enhance uptake in a proposed cardiovascular screening programme. Using an interviewing approach allowed us to elaborate on the participants' perspectives and hereby understand experiences related to their expressed preferences for facilitating participation and what they felt would challenge participation. To guide the consultation, we used a semi‐structured interview guide and interviews were recorded to ensure all details were kept for the future analysis.^5^, ^11^ The aim was to use any feedback to re‐thinking a cardiovascular screening initiative DIACAVAS.

### 2.1 Context

Participants were recruited from two general practices and two diabetes outpatient clinics in three of the five Danish Regions (the Central Denmark Region, the Region of Southern Denmark and the North Denmark Region). Prior to the study, staff group information meetings were offered.

Data for this PPI study were collected in the setting within which the participants were being monitored for their T2D and recruited for this study.

### 2.2 Contextual framework

In Denmark, equal access to health services for all residents is a health‐care cornerstone. The health‐care system offers publicly financed preventive, primary care and hospital services. All registered Danish residents receive a health insurance card provided by the Danish public authorities. Reimbursement of medicine carries some co‐payment depending on the individuals' annual use of prescription medication.^12^ In 2019, the maximum co‐payment for reimbursement medicine was DDK 4110 per year (552,45 euro).^13^ A total of 98% of the Danish population is listed with a general practitioner (GP).^14^ GPs coordinate medical care for the majority of people with T2D, whereas a minor share of these patients are followed in outpatient clinics.

All Danish residents have a personal digital mailbox provided by the Danish public authorities.^15^ Using the digital mailbox is an easy and well‐known strategy to reach the majority of the Danish population in a safe, secure and inexpensive manner. In 2019, only 4.2% of 35‐ to 64‐year‐old Danes living in the region where the DIACAVAS was offered did not receive digital post due to language difficulties and disabilities, for instance.^15^

### 2.3 Sampling strategy

Eligible participants were identified by purposive sampling of 40‐ to 60‐year‐old men and women with T2D from diabetes care settings. The inclusion criteria were being diagnosed with T2D without limitations due to diabetes duration, ability to speak Nordic languages or English and willingness to participate in the study. This approach was taken as people with lived experience of the condition being studied were considered experience‐based experts.^16^ A total of 24 patients were invited, 17 (70.8%) of whom accepted to participate. Busyness was the main reason stated for declining to participate in the study. Participants were recruited face‐to‐face either by their treatment provider or by the first author. The interviews were scheduled so as to fit the participants' availability.

### 2.4 Data collection

Interviews were conducted using an interview guide based on the scope of PPI (Table 1). We pilot‐tested the interview guide by conducting two interviews with participants meeting the study inclusion criteria. No changes to the interview guide were needed. Therefore, these interviews were included in the final PPI study.

**TABLE 1:** Interview guide

Main topics	Questions related to the main topics
The invitation	Thoughts and notes recorded while reading the invitation letter How may it be improved? How do you think that you would experience receiving such an invitation?
The screening set‐up	Thoughts and notes related to the proposed screening initiative including set‐up Does it deliver what is important to you? How may it be improved?
Participation	How may participation be facilitated?
Perspective on cardiovascular screening	Personal and in general

Data were collected from April to October 2019 by the first author. Data collection was continued until the sample met the inclusion criteria and the authors deemed that further data would not add to the analysis.^17^

Prior to the interview, the participants were asked to read and make notes to a proposed invitation letter for cardiovascular screening. An English version of the invitation is available in the supporting information. The invitation consisted of an invitation letter (Appendix S1) and some participant information (Appendix S2). The proposed invitation letter was similar to the one used in the DIACAVAS, but without illustrations of the screening examinations. The DIACAVAS screening set‐up and examination are described in detail elsewhere.^2^ Briefly, they included examination for coronary artery calcification, aortic and iliac aneurysm, atrial fibrillation, peripheral arterial disease, hypertension and hypercholesterolaemia. The screening examinations were performed at [hospital], Denmark. The principal investigators of the DIACAVAS were the sender of the invitation, which was sent to the personal digital mailbox of invitees without a pre‐booked appointment. Invitees without digital mailboxes received the invitation by standard surface mail. The DIACAVAS invitation and set‐up were approved by the Southern Denmark Region Committee on Health Research Ethics (S‐20180066) and the Danish Data Protection Agency (18/40178).^2^

When the participants read the invitation, the interviewer left the room so that they could read it undisturbed and at their own pace. Once the participants had finished reading the invitation, including making notes, they asked the interviewer to re‐enter. The time taken to read and make notes to the invitation letter ranged from 14 to 22 minutes. Subsequently, interviews were conducted. The interviews were audiotaped and transcribed verbatim by a research assistant. The length of the interviews was 8‐17 minutes, excluding the time needed to obtain informed consent. Notes were made after the interview relating to, for example important unrecorded statements and the interview setting.

### 2.5 Data analysis

We conducted an interpretive, inductive content analysis following the recommendations by Elo and Kyngas.^17^ The first author performed the analysis by first reading and then rereading the transcribed interviews to get an impression of the data. Next, units of analysis related to the research question were identified and coded by using an open coding approach. Subsequently, coded contents were compared in terms of similarities and differences to determine coded contents that could be synthesized into subcategories. This abstraction process was an iterative process moving back and forth between raw data, coded contents and subcategories. Then subcategories were grouped together based on similarities in contents into main categories. Finally, these categories and subcategories were discussed with the research team until consensus was achieved on both interpretations of data and sufficient data abstraction, but also to ensure credibility of findings.^17^

In this analysis, we used the software program NVivo, version 12 Pro (QRS International Pty Ltd), as a structural tool to underpin the analysis.

### 2.6 Researcher characteristics and reflexivity

The first author who conducted the interviews and analysis was not involved in the DIACAVAS, but has experience with cardiovascular screening in terms of designing, implementing and evaluating such initiatives. The research group consisted of multidisciplinary experts from various fields of nursing and medicine. The research group contributed with valuable perspectives to ensure the trustworthiness of the study.^17^, ^18^

### 2.7 Ethical considerations

This study was assessed by the Danish Committee of Multipractice Studies in General Practice DIACAVAS (MPU 14‐2019), which recommended the study to general practices. In Denmark, an interview study does not require ethical permission in pursuance of the current ‘Act on Research Ethics Review of Health Research Projects’ (Section 14, Sub‐section 2).^19^ Thus, this study was simply reported to and registered on the list of research conducted under the auspices of the Central Denmark Region (1‐16‐02‐331‐20). Prior to the interview, written informed consent was obtained from all participants and consent was confirmed verbally when concluding the interviews.

We recruited from different regions to ensure the anonymity of recruited study participants as well as the participating outpatient clinics and general practices.

## 3 RESULTS

In the analysis, we found that the participants viewed cardiovascular screening to be an important initiative both from a personal and social perspective with a view to preventing diabetes‐related cardiovascular disease and thereby reducing the costs associated with diabetes complications. Therefore, they suggested clarifying the relevance of the initiative for the invitees by using relational interaction and nudging. This led us to formulate two main categories conceptualized as follows: ‘making screening relevant through relation’ and ‘participation by nudging’. Selected baseline characteristics from the structured interviews are listed in Table 2.

**TABLE 2:** The participants' characteristics

Participant	Gender	Age	T2D duration (years)	Setting
1	Female	50‐54	>10	General practice
2	Female	40‐44	≥10	General practice
3	Male	50‐54	1‐4	General practice
4	Male	45‐49	1‐4	Outpatient clinic
5	Male	55‐60	1‐4	General practice
6	Male	40‐44	5‐9	General practice
7	Female	45‐49	5‐9	Outpatient clinic
8	Female	55‐60	1‐4	Outpatient clinic
9	Male	55‐60	1‐4	Outpatient clinic
10	Male	55‐60	< 1	Outpatient clinic
11	Male	55‐60	≥10	Outpatient clinic
12	Male	50‐54	≥10	Outpatient clinic
13	Male	50‐54	≥10	Outpatient clinic
14	Male	50‐54	1‐4	Outpatient clinic
15	Male	40‐44	<1	Outpatient clinic
16	Male	45‐49	5‐9	Outpatient clinic
17	Female	40‐44	1‐4	General practice

### 3.1 Making screening relevant through relation

We found that it was pivotal to establish a relation between the invitee and the sender of the invitation to make cardiovascular screening personally relevant. The wording of the invitation did not only present specific content, but also established a specific and inexpedient relationship. Using medical terminology created an impersonal professional distance to the recipient:

SLGT‐2 inhibitors? I don't understand it, no one does … maybe you could talk it over with your own doctor, because many people trust their own doctor (Participant 16).

Furthermore, the senders' academic titles distanced readers from the senders:

Professorships and PhD titles make the screening offer less attractive, without such fine titles it is more down to earth and speaks more to the general public (Participant 16).

Thus, for readers to find that cardiovascular screening is personally relevant, the invitation needs to facilitate a one‐way communicative relationship through rhetoric.

A personal relation achieved by involving the patient's usual treatment provider seemed important in facilitating screening participation. Participant 17 expressed:

I feel that it is nice that it is the same person you see … because one's own doctor and nurse, they know you, and know what issues I have with different things.

Among participants who found that the invitation was difficult to understand, the treatment providers became pivotal for participation:

Because the meaning disappears a little bit, so it has to come from the doctor … He will tell me if it is something that I should do (Participant 3).

However, not having the same treatment provider over time left participants with an impersonal relation:

I have had a lot of practitioners. The last three times I have been here, I have seen three different … it makes me feel just like a number … you don't know each other (Participant 16).

Among participants who experienced an impersonal relation, the treatment provider was not considered a diabetes management resource. Thus, we found that people with T2D need continuity and personal relationships. Therefore, it might not be beneficial for the invitees to involve shifting treatment providers in discussions about cardiovascular screening.

We found that participants expressing a strong relation to their treatment providers seemed open‐minded about being encouraged to participate through verbal information and also through the written screening material provided in the waiting rooms in diabetes care settings. In addition, it was suggested to make the relevance of cardiovascular screening clearer by highlighting the association between diabetes and CVD:

I think it is important to explain how high a percentage gets it, because it is just a bit vague, as I said, it isn't me, who gets diabetes or a heart attack, I live healthy, to say it right out that it might as well be you as one of your colleagues (Participant 5).

When a strong relation exists, information about the screening initiative is provided by a trusted treatment provider. This may enhance the patient's attentiveness when he or she receives the screening invitation.

### 3.2 Participation by nudging

In our analysis and interpretation, we found that the participants requested support to become attentive to the person‐centred relevance of participation. They suggested using nudging in the screening invitation and the screening programme to facilitate participation.

#### 3.2.1 A nudging screening invitation

Overall, the participants found that the invitation was too extensive. One participant expressed

You never get to the benefits of participating … it is lost in that there is way too much extra stuff … it does not hit the target (Participant 3).

Similarly, Participant 5 suggested amending the structure of the information to enhance focus on critical parts:

I think that one of the most important things is what you get out of participation, and it shouldn't be written in the middle of it all, it should be at the top or at the bottom, because if people don't read it, you know people just quickly scan through it, then they don't read that … I once worked with TDC (phone company), where we watched a commercial with a lot of company names, and we noticed the first and the last, all the ones in the middle we didn't notice.

Another participant suggested linking to additional information:

If you want to read more about some things, then you can click on to another document (Participant 1).

We found it important for the participants to be attentive to the benefits of participating in order for them to find that screening was personally relevant. Therefore, in our interpretation, the invitation needed to nudge attention towards the personalized benefits of participating, thereby also facilitating an informed decision about participation as ethically and legally required.

An eye‐catching invitation with illustrations was also suggested in order to nudge the invitees' attention:

The invitation is not inspiring … the text is comprehensive and would be enough for an eight‐page booklet with illustrations of the examinations (Participant 14).

Illustrations of the examinations were also suggested to clarify concerns about the CT scan, in particular:

Is it a completely closed scanner? I can't get into it then; I have claustrophobia … I tried to get into an MRI scanner twice but couldn't. If it's an open scanner then I can. Show how it looks so you can get a sense of it (Participant 7).

In this way, illustrations may also underpin achieving informed consent.

The invitation included medical terms and wordings challenging its readability. However, some participants expressed that if the screening offer was relevant to them, they just ignored such terms:

PCSK9, I don't know exactly what that is, but then atherosclerosis was mentioned ‐ then I think it might not matter what it really is … it's like, if something is in English and you don't know all the words, then you can figure it out anyway … it's not the invitation that determines whether I participate or not (Participant 8).

Moreover, participants disregarded medical terms because they thought the terms were intended to protect the research team:

When I read something like that (PCSK9), then I just read it and say, “Oh well that is how it is”, you write all about what can happen to us, you need to protect yourself and make sure you do not get in trouble with the authorities, and that is why it is not too complicated in my world (Participant 5).

In this way, the invitees' needs and preferences for a readable invitation might be set aside in order to shield the researchers from legal issues. But, overall, we found an easy‐to‐read invitation to be preferable in order to build awareness about the relevance of participating and to allow invitees to make an informed decision.

We found that the articulation and wording of cardiovascular risk in the invitation were important in determining if participants found the screening personally pertinent and for the ability of the invitation to nudge participants towards participation. In addition, we found a variation in participants' preferences for the description of cardiovascular risk. Preferences ranged from strong to soft wording:

I think you have to put more mutilation on the table, because the invitation says nothing about the alternative if you do not accept the offer. It says something about it being voluntary to participate. No forget that, you need to know that if you do not accept this offer, you are not able to find out for yourself if you have one of these diseases that we are trying to help you with. More force is needed! (Participant 16).

In contrast, soft wording was preferable to other participants:

I think it was a little hard just to write blood clots. It's the same as saying that you walk around with blood clots and we need to look into it just because you have diabetes (Participant 6).

Thus, a hard wording of the cardiovascular risk description may have psychological repercussions.

#### 3.2.2 A nudging organizational set‐up

We found different preferences for nudging in relation to scheduling of the screening appointment. One participant preferred an open invitation with self‐scheduling of the examination:

Like, when you need to make an appointment at your doctor's, you can call and make one (Participant 6).

In general, it was questioned whether online booking was favourable:

I don't know if people would take the time to use the website to make an appointment (Participant 5).

In contrast, participants who suggested that cardiovascular screening should become a routine offer recommended sending a pre‐booked appointment:

Your appointment is at 14:00 hours on December 3, where we have scheduled you for testing. If, however, you don't wish to participate, you risk your coronary artery bursting. And by the way, you are not able to look inside yourself to see if you have formed any blood clots … that's how that is! (Participant 16).

However, Participant 6 expressed concern about nudging invitees towards participation by pre‐scheduling:

It needs to be voluntary for the individual whether they want to participate … if you force an appointment on them, well then they almost don't have a choice.

In this manner, a screening invitation that includes a pre‐booking might be considered as compulsory and therefore limit the invitees' self‐determination and autonomy.

We found that the scheduling of the screening appointments needed to fit into the invitees' everyday lives in order to nudge them towards participation:

If it is scheduled during working hours, you have to take a day off or a half day. I am very convinced that people will decline for that reason … because you would much rather spend a day off with your family (Participant 7).

Moreover, experiencing a lack of acceptance from work challenged one participant's ability to follow current diabetes‐related recommendations:

This thing about four check‐ups a year. What about your workplace, you can very quickly get hit in your head with “Why do you call the ophthalmologist all the time?”. I have gotten that! (Participant 17).

In contrast, offering screening in the afternoon and evening may be a barrier for those prioritizing their family:

The period offered is not practical (2‐8 p.m.) … it is just when you pick the kids up from school and then there is sports, homework and dinner (Participant 7).

Thus, personal and working priorities might challenge the uptake in cardiovascular screening, but flexible time slots for examinations may encourage and nudge invites towards participation.

We also found that the timing of the introduction of the screening to the invitees was of importance in terms of nudging towards participation:

If I were offered it, I would rather have received it as a newly diagnosed diabetic rather than after several years … then I would probably just have said, “Well you know what, we will just do that” (Participant 17).

Later cardiovascular screening was considered riskier and therefore a motive for non‐participation:

Well, I think, at the moment when you are offered it, all of a sudden, it is made apparent that something might be wrong and you have to relate to something that is more than just eating two pills a day. I think it is bad enough to get a heart diagram, then have to get a CT scan … What if they find something, it gets more real and bigger! (Participant 17).

Offering screening at the time of diagnosis would be considered part of the diagnostic package for people with diabetes. Consequently, they would not have to decide whether to participate or not. Additionally, it was suggested to include cardiovascular screening in routinely performed diabetes controls. The following statement was given by participants who viewed cardiovascular prevention as beneficial from an individual perspective:

You have to take diabetes seriously, if you don't, the boomerang returns at some point, and it doesn't have to be something like you losing your eyesight or that a leg has to be amputated, it can also be a fatal heart attack. Because, the hidden sequelae, it is really like having dark clouds, that hang over your head, if you aren't watching out (Participant 16).

But cardiovascular screening was also perceived as beneficial from a social perspective:

I really mean that it should be mandatory! Because of the consequences, when people get this (CVD) it will be a lot more expensive for the society as opposed to tackling the issue early on (Participant 16).

Based on these statements, we interpret that cardiovascular screening is considered beneficial, and therefore participants suggested using nudging towards participation. Notably, the participants did not mention whether the DIACAVAS is effective or not, even though the invitation emphasized that the purpose of DIACAVAS was to establish whether a screening offer reduces the cardiovascular risk.

**FIGURE 1:**
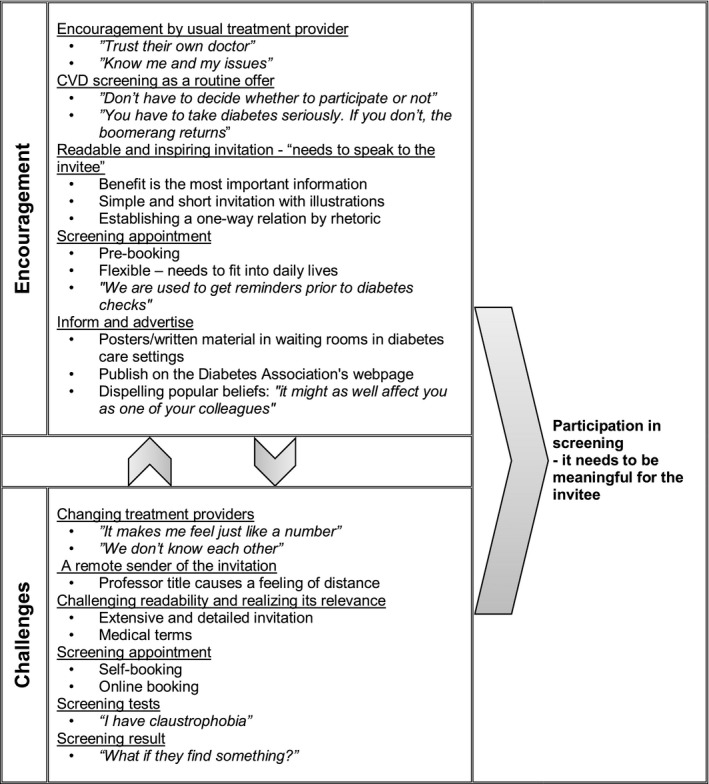
Encouraging and challenging factors involved in determining if a screening invitation is accepted

Finally, Figure 1 provides an overview of the identified encouraging and challenging factors relating to screening participation. We found interpersonal variation in what was considered as encouraging and challenging factors suggesting that a person‐centred invitation approach may be appropriate. As such, involving the usual treatment providers in recruiting is necessary.

## 4 DISCUSSION

In this study, we obtained important feedback from people with T2D and established that relational aspects and nudging are considered essential in facilitating uptake in a cardiovascular prevention initiative. In the following, we discuss the possible effects of our findings.

### 4.1 Relational interaction is essential in facilitating participation

Overall, we found that a relationship between the patient and the health‐care provider was essential in making the invitee perceive the screening as personally relevant. If people with T2D are to participate, screenings must be meaningful to them, and this may be achieved by building on a previously established relationship. According to a narrative review and synthesis by Kitson and colleagues,^20^ the relationship between patients and treatment providers is a core element of patient‐centred care across health‐care disciplines. Patient‐centred care involves, for example having and maintaining a genuine relationship, having a cohesive team of treatment providers and creating a setting in which knowledge is shared and flows freely between patient and treatment providers.^20^ Moreover, a relationship is based on a moral commitment by the treatment providers to care for patients.^21^ Accordingly, a systematic review by Harrington and colleagues found that reinforcing patient–doctor communication was beneficial for recall of information, participation and adherence to recommendations, long‐term changes in health status and lifestyle as well as for patient participation preferences in medical consultations.^22^ Communication may hence facilitate shared decision making that involves eliciting the individual's preferences.^23^ Therefore, we argue that a relationship‐centred approach is important when treatment providers encourage participation in screening because the encouragement to participate will then be based on the individuals' preferences. In Denmark, citizens are affiliated with a specific general practice. This allows for continuance in diabetes care, among others, but also provides the foundation for the establishment of a relationship between patient and GP that includes knowledge about the individual patient.

In screening, personalized prompts from health professionals have been found to be particularly effective in enhancing participation. In a randomized controlled trial among invitees to the NHS Health Check for CVD, Gidlow et al^24^ found that the uptake increased significantly from 30.9% to 47.6% among those invited by a phone call from the practice staff compared with receiving a standard invitation letter. Moreover, in an analysis of recorded invitation approaches (n = 12 048) in 30 general practices, Cook and colleagues found that the overall uptake of face‐to‐face invitation was 71.9% compared with 43% by telephone invitation and 29.5% by written invitation.^25^ This is in line with our finding that personal encouragement to participate based on a trusted relationship with the invitee is important in ensuring that the screening invitation is considered personally relevant. Similarly, a recent systematic review by Brewster and colleagues found that non‐participation in diabetes outpatient settings was related to impersonal relationships with treatment providers.^26^ Therefore, we suggest that invitees may be more attentive to a personalized invitation if its sender is a well‐known health‐care professional, for example the family's GP.

Interestingly, we found that people with T2D did not view health professionals with higher academic degrees to be a significant authority. In contrast, academic degrees were perceived as a distancing element for establishing a relational sense. Taking a sociological approach, our findings show a change in society's hierarchical structures and people's experience of authorities. Literature suggests that the widespread liberalist approaches in the Western world have challenged the conservative structures of the past and introduced human views, where people have a more individual‐centred focus.^27^

### 4.2 Nudging as a strategy for participation in screening

To our knowledge, this is the first study illuminating that invitees suggest the use of nudging in cardiovascular screening prospectively. Recently, nudging has been defined as ‘an umbrella term for deliberate and predictable methods of changing people's behaviour by modifying the cues in the physical and/or social context in which they act. It uses these cues to activate non‐conscious thought processes involved in human decision‐making. Nudging implies that none of the choices should be difficult to avoid, made mandatory, incentivized economically or socially, and made significantly more costly in terms of time or trouble’ (page 3).^28^ We found that the participants advocated use of nudging in the design of the invitation and the screening programme.

We found that the participants considered their personalized benefits of participating to be the most important information in the screening invitation. Moreover, awareness towards the benefits made participation meaningful to them. Thus, nudging the invitees' attention towards this part of the invitation was proposed, which may furthermore facilitate an informed decision when invitees decide whether to attend or not. Studies across countries and populations show that participants may have a limited understanding of the research in which they have been invited to participate. Interview studies have found that a contributing factor for declining an invitation to a cardiovascular preventive initiative is misunderstanding of its purpose or failure to understand that the offer comprised more than the routine health check at their GPs.^29^, ^30^, ^31^ Additionally, a shorter invitation letter highlighting the key aims was requested.^30^ These findings are supported by a review by de Ward and colleagues who found that clear information facilitated participation.^32^ Similarly, we found a need to simplify the proposed invitation letter. This is in accordance with a review by Camilloni et al^33^ stating that long, detailed letters may increase inequalities in participation in organized screening programmes by discouraging those with lower education levels. However, a brief information material might conflict with legal requirements.

Disquieting, we found that invitees might ignore their own needs for a readable invitation, thinking that the invitation is meant to protect the research team from litigation. Thus, the legal and moral requirements guiding the drafting of study information may not always lead to an informed decision as intended in research and outlined in the Declaration of Helsinki and by the Council for International Organizations of Medical Sciences.^34^, ^35^ Moreover, an invitation to a screening needs to be understandable.^36^, ^37^ These concerns need to be addressed in future research. We argue that the identified encouraging and challenging factors relating to participation may also impact the invitees' ability to make an informed decision when facing a screening invitation.

In the invitation tested here, the wording used to describe cardiovascular risk was found to be important for the acceptability of nudging invitees towards participation. However, the preferences of the risk description were contradictory as they ranged from soft to hard word wording. Consequently, the description of the cardiovascular risk might need to be worded softly in order to minimize harm, as also emphasized in the screening criteria outlined by the health authorities.^36^, ^37^

A pre‐scheduled appointment is known to increase participation in screening for breast cancer.^38^ Similarly, we found that our participants recommended offering a pre‐scheduled appointment, although concern was expressed about the risk of violating the invitees' autonomy. However, legally, a screening invitation must stress that participation is optional and that whether the invitation is accepted or not is without consequences for the invitees' current treatment. Thus, pre‐scheduling seems an acceptable intervention that is in accordance with the definition of nudging as the choices are neither difficult to avoid nor made mandatory. In cardiovascular population‐based screening, pre‐scheduling has produced a 74% uptake.^39^, ^40^ In a later publication, one of these studies has reported the specific uptake for invitees with diabetes and found diabetes to be significantly associated with non‐participation.^3^

### 4.3 Ethical aspects of nudging in screening

We found that the participants advocated using nudging in cardiovascular screening offers. This should be seen in the light of the fact that they considered cardiovascular screening an important preventive initiative and that some preferred not to face the decision of whether they should participate or not. Similarly, Bowling and Ebrahim^41^ found that some patients did not want to be involved in the decisions about their treatment due to a lack of knowledge and experience and for fear that they might make the wrong decisions. Even so, most patients wanted treatment providers to understand their perspectives, even if they did not want to make the final decision themselves. The moral commitment in patient‐centred care means that treatment providers are obliged to nudge patients towards participation in accordance with the individual's preferences.

If people with diabetes advocate nudging in screening, they either think that nudging does not violate their autonomy, or they believe that nudging transgresses their autonomy but accept this owing to the positive effects of nudging. However, as the hidden sequelae of diabetes were described by participants as ‘it is really like having dark clouds that hang over your head’, people with diabetes may have different preferences in relation to nudging than the general population.

### 4.4 Discussion of method

We used PPI in recognition of the low uptake in the DIACAVAS pilot study. This study contributed with important information about the relevance of offering cardiovascular screening and how to facilitate participation in forthcoming preventive initiatives. However, it may be favourable to also incorporate PPI in re‐thinking process of the DIACAVAS study and to invite people with T2D to become members of advisory or management groups, for instance^42^; an initiative which is in line with one of our key findings; the relational component.

We performed qualitative research interviews which rely on an active process in which the interviewer and interviewee produce knowledge through their relation within a certain context.^43^ We used individual interviews to gain profound and personal insights^8^ and to ensure that each participant was active and felt free to share their views.^44^, ^45^ Interviews are preferable when the research question aims to explore the interviewee's perspective of a phenomenon rather than generating generalizable understandings from large groups of people.^46^ Moreover, interviews is a recommended approach in PPI.^11^ Alternatively, workshops may be favourable as they allow participants to inspire each other and engage in discussions based on their personal experiences, attitudes and ideas. Workshops may also be a suitable manner to prioritize new initiatives facilitating participation.^47^

The analytical approach was based on content analysis, the purpose of which is to provide knowledge and new insights about a complex phenomenon while also providing a practical guide to action.^17^, ^48^

The study participants were characterized by wide socioeconomic variation from secondary‐level to tertiary‐level education and their working status varied from being outside the labour market (retired, unemployed) to being employed or self‐employed. However, people of a non‐Western origin were not represented. Given the limitations related to the demographic characteristics of the participants, it would be interesting to explore possible ethnic differences, which is a potential area for future research.

Our findings may be disease‐specific as people with diabetes may have special preferences for nudging due to the severity of the diabetes diagnosis.

## 5 CONCLUSION

Support for cardiovascular screening among people with T2D appears to be widespread. To enhance the uptake in such initiatives, focussing on recruitment is essential. To facilitate participation, we found that people with T2D emphasized the relation with their usual health‐care professionals. Therefore, any screening offer should be provided in collaboration with these health‐care professionals. Moreover, nudging was advocated to facilitate participation, particularly in relation to the screening set‐up and invitation. Furthermore, the written invitation needs to be prepared thoroughly and in collaboration with the invitees to ensure that the text is easy to read and understand.

Overall, this study contributed with knowledge on how to promote participation by involving health professionals and using nudging in accordance with the preferences of people with T2D. This knowledge is valuable for researchers, policymakers and ethicists as it enhances our understanding and assessment of the ethical appropriateness and public approval of nudging in cardiovascular screening.

Finally, participation is a general concern, not only in relation to cardiovascular prevention. Our findings might be applicable to other preventive services targeting people with T2D.

## CONFLICT OF INTEREST

The authors declare that there are no conflicts of interest.

## AUTHOR CONTRIBUTIONS

MD, AD and JS designed the study. MD collected field data, conducted the analysis and drafted this manuscript. All authors contributed with constructive criticism during the design of the study, analysis of the data and preparation of the manuscript. All authors have read and approved the final manuscript.

## Supporting information

Appendix S1

Appendix S2

## Data Availability

The entire transcribed interviews used in this study are not publicly available. But minor anonymized parts are available from the corresponding author upon a reasonable request. This is to protect and maintain participants' anonymity and confidentiality.
