# Supplementary material for: Involving people with type 2 diabetes in facilitating participation in a cardiovascular screening programme
Source: Health Expect. 2021 Mar 24;24(3):880–91. doi: 10.1111/hex.13228 (PMC8235888; doi:10.1111/hex.13228)
Supplement: Supplementary file 1 — Appendix S1 [file HEX-24-880-s002.pdf]

Appendix 1. Proposed invitation letter

Dear

We hereby ask you if you would like to participate in the scientific trial coined DIACAVAS.

We have contacted you because you have diabetes, and we wish to study if you have an increased risk of blood clots. If we find that this is the case, we will offer you preventive treatment.

You will find more information about the trial in the enclosed letter. If you have any questions about the trial, feel free to call/send an e-mail to the physicians in charge of the project - please see the enclosed participant information.

If you wish to participate or would like learn more about the trial, please schedule an appointment within four weeks at https: https://diacavas.rsyd.dk/

Your personal password is: XXXXX

Alternatively, you may call or send an email to:

Screening Secretariat, Odense University Hospital
Phone +45 XXXXXX (Marianne) or +45 XXXXXX (Henriette)
Opening hours: Daily from 9AM to 2PM

The conversations are held in Odense on Tuesdays and Thursdays, 2 p.m.-8 p.m. If you decide to participate, you will be examined immediately after the conversation.

Conversations are held at the Cardiac CT scanner in the basement. Go to Entrance 27 in the entrance hall of the OUH Tower Block. From here, follow the black arrows which will lead you to the Cardiac CT scanner. Take a seat there. We will call you when it is your turn.

We will invite you once more, but if you are not interested in participating, we would be grateful if you would let us know.

Kind regards

Axel Diederichsen
Professor, Consultant, PhD
Department of Cardiology B
Odense University Hospital

Jan Frystyk
Professor, Consultant, PhD
Department of Endocrinology M
Odense University Hospital

Jes Lindholt
Professor, Consultant, MD, PhD
Cardiac, Thoracic and Vascular Surgery T
Odense University Hospital
