# Supplementary material for: Involving people with type 2 diabetes in facilitating participation in a cardiovascular screening programme
Source: Health Expect. 2021 Mar 24;24(3):880–91. doi: 10.1111/hex.13228 (PMC8235888; doi:10.1111/hex.13228)
Supplement: Supplementary file 2 — Appendix S2 [file HEX-24-880-s001.pdf]

Region of Southern Denmark

Appendix 2. Proposed written participant information

DIAbetic CArdioVAscular Screening and intervention trial – DIACAVAS

Danish title: Forebyggelse af hjertekarsygdom hos personer med sukkersyge, en screeningsundersøgelse

Introduction

We hereby invite you to participate in a health science screening trial on cardiovascular disease. We contact you because you have diabetes, and cardiovascular disease is one of the most frequently occurring problems among people with diabetes. A healthy lifestyle is recommended to reduce the risk, but this alone is not always sufficient.

What is the aim of the study?

We offer 16,500 men and women aged 40-59 years with diabetes a cardiovascular screening. All 16,500 are selected at random by data extraction from the Danish Prescription Register, and you are one of these people. If we find disease precursors, we will offer you preventive treatment. The aim is to study if preventive treatment may reduce your risk of cardiovascular disease.

What is the study about?

We will perform a CT scan that allows us to see any hardening in your coronary arteries and to establish any signs of incipient aortic disease. Additionally, we will use blood pressure measurements to study the blood supply in your legs.

1. **A brief talk about your state of health**
   - including if you are a smoker, if you take any medicines, if you have been ill, etc.
2. **Measurement of height, weight, waist and hip circumference, pulse and blood pressure in the arms and legs**
3. **Blood and urine samples and biobank**
   The blood sampling requires a prick in the arm. We will measure your cholesterol levels, blood sugar, kidney function and perform some research analyses concerning the causes and treatment of hardening of your arteries. We will measure albumin levels in urine. We will also store a blood sample (40 ml blood) and a urine sample (10 ml) in a research biobank.
4. **CT scan of the heart and aorta**
   This is an X-ray examination to measure the amount of hardening in your coronary arteries, the size of your aorta and assess your heart rhythm. It only takes a few seconds to record the images.

With the approval of the Danish Data Protection Agency, we want to store any excess blood and urine so that we can do additional tests as new knowledge about hardening of the arteries emerges. The samples will normally be stored for 10 years before being destroyed. You can always contact the physician in charge of the project and ask to have your material destroyed.

Region of Southern Denmark

Benefits of participating?

We offer to test if you have incipient cardiovascular disease, but do not screen for other conditions like cancer. We expect that one in every three participants will be healthy, whereas the rest may have incipient cardiovascular disease. If you are among the latter, we will assess your current treatment and, if needed, offer you additional medications: against thrombosis (aspirin), against hardening of the arteries (atorvastatin and possibly PCSK9 inhibitors), against diabetes (possibly a GLP-1 agonist, SGLT-2 inhibitors) and healthy lifestyle advice. If your aorta is enlarged and on suspicion of atrial fibrillation or cardiac insufficiency, we will offer you follow-up tests by a specialist. Treatment is expected to reduce the risk of cardiovascular disease by 30% and death by 10%.

Afterwards, we conclude your course at a standard check-up with your GP or at the hospital and send them a summary of your results. If needed, we will offer you another screening examination every three years. If you do not want to participate in the study, simply continue attending your normal check-ups with your GP as usual.

Are there any inconveniences/risks for me as a participant?

If we find incipient hardening of the arteries, you will be offered the treatment described above. This is the standard treatment, and your risk of side effects as a participant is not larger than it will otherwise be. The CT scan produces a modest radiation dose, corresponding to 2-4 milli Sievert (mSv). This is similar to the background radiation from dirt, air, food and housing of 3 mSv per year in Denmark. The radiation dose will increase your risk of cancer by 0.02%, which should be seen in relation to the general 25% risk of developing a cancer in Denmark. Thus, the resulting total cancer risk would be 25.02% If the screening study is repeated, the total cancer risk will be increased to 25.03% as control scans are less comprehensive.

How long does the examination take?

Please fill in the enclosed questionnaire ahead of the examination and bring it with you. Apart from this, no preparation is needed.

When you arrive, we will inform you of the examination and you will talk briefly with a project staff member. Among others, you will have your pulse and blood pressure measured, and have blood samples taken. Afterwards, you will be examined in the CT scanner. We expect the whole process to take less than one hour.

Why is it important that you participate in the examination?

It is being considered to offer this screening examination to the entire Danish population, but before doing so, we need to know its effect and price.

If you do not respond to this letter, we will send you a second invitation in writing.

The results of the trial will be published in scientific journals.

Consent

You may bring your spouse or a friend for the information session about the trial. After we have given you written and oral information, you may either agree immediately to participating or ask for a reflection period of at least 24 hours. If you do not want to participate, you will not subsequently be excluded. You may withdraw your consent at any time, and as the screening is not standard in Denmark, this will have no consequences for you.

Region of Southern Denmark

Person-sensitive information

If you chose to participate in the trial, you also agree that the physicians in charge of the project may access your patient record as needed to collect information about the medicine you are taking and the conditions you have, etc.The information we obtain about you from your patient record and from the examinations will be used for research exclusively, and will be pseudo-anonymised to ensure that it will not be possible to identify you or any other individuals.

Funding

The trial is a joint initiative counting Odense University Hospital, Copenhagen University Hospital (Rigshospitalet) and the hospitals of Nykøbing Falster, Svendborg, Vejle, Åbenrå, Esbjerg, Silkeborg and Hjørring. We will seek funding from private foundations like the Novo Nordisk Foundation, the Alfred Benzon Foundation and the Danish Heart Foundation, but also from the Independent Research Fund Denmark and the research funds at Odense University Hospital and the Danish regions.
The physicians in charge of the trial are in no way associated with the funders.
As a trial participant, you will receive no fee or mileage compensation. If you are offered supplementary medical treatment, you will need to buy the medicines at the pharmacy - like you normally do.

For additional information, we enclose the following material:

Leaflet: "Før du beslutter dig" (In Danish language: Before you decide) published by the Regional Committees on Health Research Ethics for Southern Denmark

Contact information

If you have any questions, feel free to write an e-mail or call one of the physicians responsible for the project:

Kind regards

Axel Diederichsen
Professor, Consultant, PhD
Department of Cardiology B
Odense University Hospital
Mobile xxxx, e-mail: xxxx

Jan Frystyk
Professor, Consultant, PhD
Department of Endocrinology M
Odense University Hospital
Mobile xxxx, e-mail: xxxx

Jes Lindholt
Professor, Consultant, MD, PhD
Cardiac, Thoracic and Vascular Surgery T
Odense University Hospital
Mobile xxxx, e-mail: xxxx

DIACAVAS
A screening and intervention study
